# The Potential Role of the ZIKV NS5 Nuclear Spherical-Shell Structures in Cell Type-Specific Host Immune Modulation during ZIKV Infection

**DOI:** 10.3390/cells8121519

**Published:** 2019-11-26

**Authors:** Min Jie Alvin Tan, Kitti Wing Ki Chan, Ivan H. W. Ng, Sean Yao Zu Kong, Chin Piaw Gwee, Satoru Watanabe, Subhash G. Vasudevan

**Affiliations:** 1 Program in Emerging Infectious Diseases, Duke-NUS Medical School, Singapore 169857, Singapore; 2 Department of Microbiology and Immunology, National University of Singapore, 5 Science Drive 2, Singapore 117545, Singapore; 3 Institute for Glycomics, Griffith University, Gold Coast Campus, Queensland 4022, Australia

**Keywords:** flavivirus, Zika virus, NS5 protein, nuclear localization, inflammation, innate immunity

## Abstract

The Zika virus (ZIKV) non-structural protein 5 (NS5) plays multiple viral and cellular roles during infection, with its primary role in virus RNA replication taking place in the cytoplasm. However, immunofluorescence assay studies have detected the presence of ZIKV NS5 in unique spherical shell-like structures in the nuclei of infected cells, suggesting potentially important cellular roles of ZIKV NS5 in the nucleus. Hence ZIKV NS5′s subcellular distribution and localization must be tightly regulated during ZIKV infection. Both ZIKV NS5 expression or ZIKV infection antagonizes type I interferon signaling, and induces a pro-inflammatory transcriptional response in a cell type-specific manner, but the mechanisms involved and the role of nuclear ZIKV NS5 in these cellular functions has not been elucidated. Intriguingly, these cells originate from the brain and placenta, which are also organs that exhibit a pro-inflammatory signature and are known sites of pathogenesis during ZIKV infection in animal models and humans. Here, we discuss the regulation of the subcellular localization of the ZIKV NS5 protein, and its putative role in the induction of an inflammatory response and the occurrence of pathology in specific organs during ZIKV infection.

## 1. Introduction

Flaviviruses are small, enveloped RNA viruses that make up one genus in the *Flaviviridae* family of viruses. Many of these flaviviruses, including dengue virus (DENV), West Nile virus (WNV), yellow fever virus (YFV) and Zika virus (ZIKV), are medically important and cause human disease. There is currently no antiviral therapeutics available for clinical use against these viruses. ZIKV was initially identified as a rather innocuous member of the flavivirus genus in 1947 [1,2], and was only highlighted as a threat during the 2015 epidemic in the Americas [3,4,5] due to the correlation of ZIKV infection with severe neurological pathologies. Like many of the other flaviviruses, ZIKV is transmitted from human-to-human via the *Aedes* mosquitoes, although horizontal human-to-human transmission through sexual intercourse has also been identified as a unique mode of transmission of ZIKV. 

The flavivirus genome is made up of a single-stranded positive sense RNA of approximately 11,000 nucleotides that encodes an ~3400 amino acid residue polyprotein precursor. This polyprotein is post-translationally cleaved into three structural proteins (capsid, pre-membrane/membrane, and envelope) and seven nonstructural (NS) proteins (NS1, NS2A, NS2B, NS3, NS4A, NS4B, and NS5). Flavivirus replication has been rigorously studied, and shown to require massive intracellular membrane redistribution to enable efficient replication within the cytoplasm of the host cell [6,7,8]. The membrane reorganization creates structures that are visualized by electron microscopy as vesicle packets within which all the viral NS proteins form the virus replication complex (RC) with host proteins that have not been fully characterized [9]. Flavivirus RNA replication occurs exclusively in these RCs within cytoplasmic membrane vesicles, where the virus genome can safely replicate out of reach of the host immune system [6,10,11,12]. 

The non-structural protein 5 (NS5) is the largest and most conserved protein encoded by the members of the flaviviruses [13,14], and plays crucial enzymatic roles during virus RNA replication as part of the virus RC. Apart from these functions, flavivirus NS5 has other viral and cellular roles during virus infection, including the modulation of the host immune response. Interestingly, the subcellular localization of NS5 proteins is different amongst the flaviviruses when examined by immunofluorescence assays, as they have been found in the nucleus and/or cytoplasm [15,16,17,18,19,20,21,22,23,24]. In particular, the observation of these flavivirus NS5 proteins in the nucleus suggests that NS5 plays important cellular and virus roles there, despite its major virus enzymatic role in virus RNA replication in the cytoplasm. In the case of ZIKV, most, if not all, of the detected NS5 protein form discrete spherical shell-like structures in the nucleus [21,25,26]. Taken together, the localization and distribution of ZIKV NS5 has to be tightly regulated for it to fulfill all its different roles during virus infection, but this is not well elucidated at the moment. As with many other flavivirus NS5 proteins, the significance of its localization to the nucleus on its cellular functions during infection is not known. In the first part of this review, we will use this recent demonstration of unique ZIKV NS5 nuclear localization as a springboard to discuss the regulation and function of the sub-cellular localization and, in particular, the nuclear role of the flavivirus NS5 protein.

During virus infection, the host responds by triggering a variety of signaling cascades, especially those involved in the immune response. The activation of these pathways such as the antiviral and pro-inflammatory signaling pathways leads to the induction of an antiviral state that helps the host repress virus replication and clear the virus [27]. In turn, viruses have developed mechanisms to modulate and impair these responses, and often utilize multiple strategies to target these signaling cascades. Flaviviruses do so by encoding multiple virus proteins that can antagonize or suppress the host immune response [28], as multiple DENV [29,30,31] and WNV NS proteins [32,33,34] have been described to repress antiviral pathways such as the type I interferon signaling pathway. ZIKV is no exception, as its NS2A, NS2B, NS4A and NS4B proteins have been described to antagonize the type I interferon pathway by targeting distinct components of the signaling cascade [35]. The flavivirus NS5 protein is another major player in this process [36,37], as it has been characterized to antagonize type I IFN signaling at multiple points [26,31,38,39]. Like its other flavivirus NS5 counterparts, ZIKV NS5 is able to inhibit the host antiviral response [25,26,39,40], although the significance and contribution of its nuclear localization to this process is not known.

Besides antagonizing the host antiviral response, ZIKV infection induces the transcriptional activation of pro-inflammatory genes [21,25]. Intriguingly, this activation is only observed in certain cell types such as neural and placental cells, but not in others such as liver cells. While the detailed mechanism of this cell type-specific activation is not known, ZIKV NS5, and its nuclear localization, has been shown to contribute to this transcriptional activation, as the expression of ZIKV NS5 alone in neural cells is able to trigger a pro-inflammatory transcriptional response. In the second part of this review, we will discuss the possible role of ZIKV NS5′s subcellular localization in the modulation of the host immune response during ZIKV infection, and the pathological outcome in specific tissue during infection. 

## 2. Subcellular Localization of Flavivirus NS5

**Figure 1:**
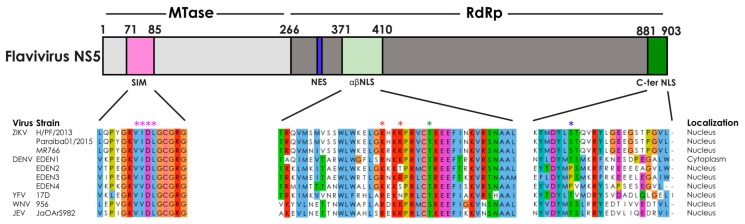
Sequences involved in the regulation of the subcellular localization of flavivirus NS5. (Top) The flavivirus NS5 consists of two functional domains, the methyltransferase (MTase) and the RNA-dependent RNA polymerase (RdRP). The regions involved in its subcellular localization (SIM, NES, αβNLS and C-ter NLS) are also denoted. (Bottom) Amino acid sequence alignment of the denoted regions of NS5 from various flaviviruses showing the sequence conservation using the Clustal X color scheme. The aligned NS5 regions contain the SIM (left), αβNLS (nuclear localization signal) (middle) and C-terminal NLS (right) sequences. The SIM motif is indicated with pink asterisks, two residues mutated in the Zika virus (ZIKV) NS5 NLS mutant are indicated with a red asterisk, CK2 phosphorylated threonine residue is indicated with a green asterisk, and the critical C-terminal NLS residue is indicated with a blue asterisk. The predominant subcellular localization of each NS5 protein is indicated to the right of the alignment. The numbering of amino acid residues is based on H/PF/2013. The virus strains in the alignment and GenBank accession numbers are as follows: H/PF/2013 (KJ776791), Paraiba01/2015 (KX280026), MR766 (LC002520), EDEN1 (EU081230), EDEN2 (EU081177), EDEN3 (EU081190), EDEN4 (GQ398256), YFV (NC 002031.1), WNV (NC_001563.2) and JEV (NC_001437.1).

The flavivirus NS5 protein bears two well-characterized enzymatic activities that are essential for virus replication: an N-terminal methyltransferase (MTase) function and a C-terminal RNA-dependent RNA polymerase (RdRp) activity (Figure 1). Additionally, it is also believed to contain a guanylyltransferase activity required for RNA cap formation [41,42]. While these flavivirus functions occur in the cytoplasm of infected cells, the NS5 protein from several flaviviruses are already present in the nucleus of the infected cells from earliest detectable time points post-infection [15,16,17,18,19,20,21,22,23,24] (Figure 1). Immunofluorescence assay studies have shown that >95% of fluorescence signal associated with NS5 can be located in the nucleus from around 24 h post-infection. Flavivirus NS5 subcellular localization has been best studied in the context of dengue virus (DENV) [15,17,18,19,20,22,23,43], trace amounts of DENV2 NS5 have been found co-localising with dsRNA, in addition to the NS5 detected in the nucleus [23]. Other flavivirus NS5 such as Zika virus (ZIKV), the main subject of this review, Japanese encephalitis virus (JEV), yellow fever virus (YFV) and West Nile virus (WNV) [16,21,24,44] have also been reported to accumulate in the nucleus. The regulation and function of the sub-cellular localization NS5 has to be tightly controlled in order for it to perform all its functions in the cell, but this is not well elucidated. 

The NS5 of flaviviruses is the largest virus-encoded protein of ~900 amino acids in size (Figure 1). At over 100kDa, the flavivirus NS5 protein can only enter the nucleus at a slow rate via passive diffusion [45], and hence will need to rely on active transport through the nuclear pore complex for efficient entry into the nucleus. In order for a protein to be transported in and out the nucleus, it needs to contain a nuclear targeting sequence that is recognized by its specific receptior from the karyopherin superfamily of proteins. In the case of nuclear import, the protein contains a specific sequence of amino acids (typically arginine and lysine) called the nuclear localization signal (NLS), and this is recognized by the importin family of proteins that mediate the movement of proteins from the cytoplasm into the nucleus [46,47].

Previous studies have revealed two highly conserved NLS sequences in the flavivirus NS5 proteins (light green in Figure 1) [15,22]. The αβNLS region was initially characterized to be the NLS of DENV serotype 2 (DENV2) NS5 [20,43] in studies involving the fusion of the NS5 NLS region to β-galactosidase and in vitro nuclear localization strategies. However the subsequent availability of a NS5-specific antibody [48] and a DENV2 infectious cDNA permitted an extensive structure-based assessment of the predominantly nuclear DENV2 NS5 and predominantly cytoplasmic DENV1 NS5, leading to the surprising finding that the previously characterized αβNLS sequence of DENV2 NS5 could not localize a truncated NS5 protein to the nucleus [23]. Instead, the NLS sequence within the C-terminal 18 residues region of DENV2 NS5 (dark green in Figure 1 and referred to as C-ter NLS) was sufficient to direct NS5 to the nucleus. Moreover the introduction of a single substitution at NS5 amino acid position 884 (blue asterisk in Figure 1) from Proline (as found in DENV2, 3 & 4) to Threonine (as found DENV1) into the DENV2 infectious clone produced a recombinant DENV mutant virus that replicated like the wild-type virus, yet showed a predominantly cytoplasmic NS5 somewhat similar to DENV1 NS5 sub-cellular localization [23]. This study showed that the sequence motif recognized by nuclear transport proteins is present in DENV1 NS5, but its conformation needs to be accessible in order for efficient transport to occur. Collectively this led to the hypothesis that NS5 nuclear localization is independent of viral RNA replication and its impact on pathogenesis must be linked to its non-enzymatic functions. 

Nuclear export can also play a role in the subcellular localization of proteins as has been reported for several virus proteins [24,49,50,51], including flavivirus NS5 which has a conserved nuclear export sequence (NES) (Figure 1). But broader validation studies are needed to evaluate the functional role of nuclear export in flaviviruses, especially in the context of ZIKV NS5 which forms supramolecular spherical shell-like structures in the nucleus [21].

The role of nuclear NS5 has been investigated for several flaviviruses [20,22,49]. However the ZIKV NS5 protein was found to form discrete spherical shell-like nuclear structures, which is distinct from the diffuse distribution throughout the nucleus noted for other NS5 proteins [16,23]. But the significance of these spherical shell-like structures is not known, and further studies have to be done to elucidate this.

### 2.1. The Nuclear Localization Signal Sequence of ZIKV NS5

An examination of the NS5 sequences from a variety of flaviviruses reveal that the C-ter NLS sequence in DENV NS5 (dark green in Figure 1) is not conserved in ZIKV NS5. In contrast, the αβNLS region (light green in Figure 1) is highly conserved amongst several members of the flavivirus genus. Hence the functional NLS in the context of the full-length ZIKV NS5 protein appears to be within this αβNLS region involving residues 388–393, and this was confirmed by mutagenesis studies in which a K390A/R393A double mutation (red asterisks in Figure 1) was sufficient to alter the subcellular localization of ZIKV NS5 from being in the nucleus to being in the cytoplasm [21]. In addition, the spherical shell-like structures formed by wild type ZIKV NS5 in the nucleus is not observed, as the ZIKV NS5 NLS mutant is diffusely localized in the cytoplasm. This suggests that the formation of nuclear spherical shell-like structures that colocalize with host proteins such as STAT1 and importin-α [21] may require host nuclear proteins and/or be regulated by additional cellular processes. 

### 2.2. Other Cellular Processes Regulating the Subcellular Localization of Flavivirus NS5

While the crucial role of NLS sequences in the nuclear transport process of flavivirus NS5 proteins has been demonstrated through site-specific mutagenesis, additional cellular processes such as post-translational modifications (PTMs) have also been shown to play a role in the regulation of the subcellular localization of cellular proteins [52]. In fact, correct localization of cellular proteins has also been shown to be important for proper function of these proteins [53,54]. In the case of flavivirus NS5 proteins, YFV [16] and DENV NS5 [43,55] are phosphorylated and localized into the nucleus, with a hyperphosphorylated form of DENV NS5 being associated with nuclear localization [19]. Interestingly the study by Kumar et al. showed that mutations of the residues adjacent to the highly conserved threonine residue in the putative CK2 phosphorylation site of DENV NS5 can alter its subcellular localization, presumably since the C-ter NLS of DENV2 NS5 is structurally part of the thumb subdomain close to the CK2 site [23]. Phosphorylation, in particular CK2 phosphorylation, of sites near NLS sequences has been utilized by multiple viruses to regulate localization of their virus proteins [56,57,58,59,60]. We note that this CK2 site is also conserved in ZIKV NS5 as well as other flaviviruses but the exact order or physiological setting that causes the phosphorylation of the CK2 site to influence sub-cellular localization is unclear. Nevertheless, the proximity to the CK2 residues and the potential phosphorylation of the conserved threonine residue (green asterisk in Figure 1) might add an additional layer of regulation to ZIKV NS5′s subcellular localization and warrants a revisit in the context of DENV2 NS5 in the light of the discovery of its C-terminal NLS [23]. Interestingly, the DENV4 NS5 NLS at the C-terminal region might be regulated by phosphorylation, based on sequence prediction although this is yet to be evaluated experimentally [23,61].

Another PTM that regulates cellular nuclear localization involves the small ubiquitin-like modifier (SUMO) proteins that are a family of small proteins closely related to ubiquitin. Like ubiquitin, it can be reversibly attached to other cellular proteins via a series of enzymatic reactions. Modification of the SUMO target protein or SUMOlyation can functionally alter the protein by inducing a conformation change, or hindering or creating binding sites for interaction with other proteins [62]. SUMOlyation of a variety of proteins including CtBP1, CREB, Mdm2, KLF5 and Lipin-1α [63,64,65,66] have been associated with nuclear localization. The flavivirus NS5 protein has a highly conserved SUMO-interacting motif (SIM) within its methyltransferase (MTase) domain [67] (pink in Figure 1), and this SIM has been demonstrated to be necessary for the SUMOlyation of the DENV NS5 protein [68]. But it is not known what role SUMOlyation plays in the subcellular localization of the NS5 protein. Further studies need to be performed to understand the contribution of the SIM domain and SUMOlyation process to the regulation of ZIKV NS5 subcellular localization, and how that relates to ZIKV pathogenesis.

Overall it can be summarized that the regulation of the subcellular localization of flavivirus NS5 proteins is complex and multifaceted, and a thorough evaluation of this process is needed in future studies. Although there may be broad similarities in the mechanism of subcellular localization of flavivirus NS5, the consequences in the context of pathogenesis may be different in line with the proposed divergent evolution of flaviviral NS5 proteins based on their conformational flexibility [69]. The location of the NLS sequence in DENV2 NS5 appears to be clearly in the C-terminal part of the protein (residues 883–900) [23] whilst that for ZIKV NS5 is within the αβNLS region [21]. However, both these sequences are structurally in the thumb sub-domain of NS5 [69,70,71,72,73,74] and will be located close to the conserved CK2 phosphorylation site. One possible scenario is that SUMOylation of NS5 could result in increased dynamics of the region around the thumb subdomain that may lead to its engagement with host proteins. In this context the structural studies of DENV NS5 using hydrogen-deuterium exchange (HDX) mass-spectrometry analysis [69] showed that the thumb subdomain appears to be more flexible and consistent with its identification as a “hot spot” for protein-protein interaction. It is likely that flavivirus NS5 proteins are separated into distinct sub-populations by their subcellular localization during various stages of flavivirus infection, and that these subpopulations are dynamic and fluid as the post-translational modifications alter the relative proportion of flavivirus NS5 in each subpopulation. Detailed investigation of this would require the reverse engineering of the virus to identify mutations that could partition the various functions without impacting viral RNA replication. This has not yet been achieved in the context ZIKV NS5 studies and suitably reverse engineered viruses would be needed to get a better understanding of these non-enzymatic functions of NS5. However the thumb sub-domain region is also critical for RNA replication function and involves direct interaction with RNA, so it might be challenging to examine the consequence of the site-specific mutations in the αβNLS in the context of infectious cDNA clones. Nevertheless, our hypothesis, at least in the context of ZIKV NS5, is that the nuclear spheres that form in the infected cells can result in different innate immune modulation within different tissues extrapolated from the demonstration in liver Huh7 cells and neural LN229 cells [21]. It can be envisaged that the ZIKV NS5 nuclear bodies sequester different subsets of host proteins at different time points in different tissues, and this point would be further elaborated below.

## 3. Cell-Type-Specific Modulation of the Host Immune Response by ZIKV and ZIKV NS5

**Figure 2:**
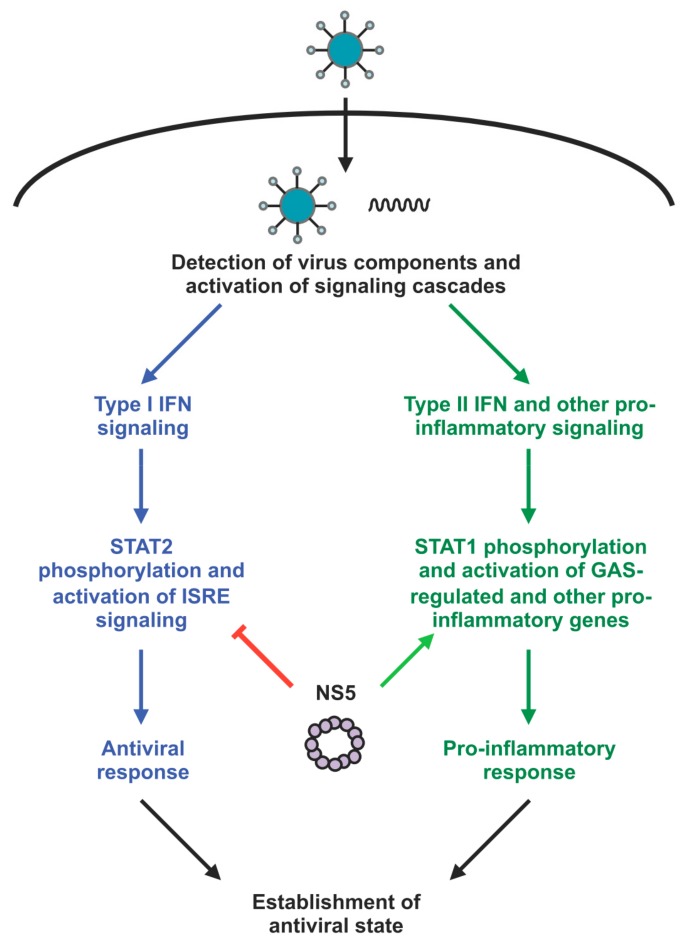
Modulation of host immune response by ZIKV NS5. Virus infection leads to the activation of host signaling cascades in response to the recognition of virus components such as RNA and protein. The type I IFN signaling pathway is one of the many pathways that mediate the host antiviral response. Type I IFN induces the phosphorylation of STAT2 and activation of interferon-stimulated response element (ISRE)-regulated genes, and this signaling cascade is targeted by ZIKV NS5 through the degradation of STAT2. Type II IFN and other pro-inflammatory molecules induces the pro-inflammatory responses through STAT1 phosphorylation and activation of interferon-gamma activated site (GAS)-regulated and other pro-inflammatory genes. ZIKV NS5 can activate the transcription of pro-inflammatory genes in a cell type-specific manner, but the mechanism is not known.

In response to virus infection, the host activates or suppresses various signaling pathways. Many of the pathways activated upon virus infection are part of the host immune response. The antiviral and pro-inflammatory signaling cascades are two major pathways of this host immune response, and work to suppress virus replication and clear the virus through the establishment of an antiviral state in the cell (Figure 2). Thus all viruses have also evolved to subvert the host immune response. The flaviviruses utilize a variety of strategies to evade, antagonize and suppress the host immune response to virus infection. One of the major pathways activated as part of the host antiviral response is the type I interferon (IFN) response. The production of type I IFN is triggered by host sensors that detect the presence of virus components such as nucleic acid. Type I IFN then activates a number of interferon-stimulated genes (ISGs) that mediate the antivirus response and establishment of an antiviral state [36,37]. Like its other flavivirus NS5 counterparts, ZIKV NS5 has been well characterized to inhibit the host antiviral response [25,26,39,40] (Table 1). But the mechanisms involved including whether ZIKV NS5′s nuclear localization plays a role have not been fully elucidated.

While the typical countermeasure of the virus to the host antiviral response is one of suppression, the relationship between the virus and the host inflammatory response is more complex. The host pro-inflammatory response is a double-edged sword. A controlled response can suppress virus replication and promote virus clearance. In contrast, an excessive response can actually lead to pathogenesis as in the case of DENV and influenza virus infections [78]. In addition, some viruses such as human cytomegalovirus (CMV) and human immunodeficiency virus (HIV) [79,80] have their replication enhanced through the induction of a host inflammatory response. ZIKV infection can induce a pro-inflammatory response in cell culture models in a variety of cell types from different organs, including the brain, testes, placenta and eyes [21,25,76,77,81] (Table 2). Interestingly, many of these organs are also sites of virus replication, persistence and pathology in multiple animal models [82,83,84,85,86,87]. Virus persistence has also been detected in human samples from these tissues, with the brain being an example of a known site of pathology for ZIKV infection [88,89,90,91,92]. Despite all these studies, the relationship between the host inflammatory response, and ZIKV replication, persistence and pathology is still unclear. Hence further and more comprehensive studies need to be performed to further our understanding of this complex relationship. In addition, it is unclear what aspects of ZIKV infection leads to the induction of this inflammatory response, although the expression of ZIKV NS5 alone appears to be sufficient to induce a pro-inflammatory transcriptional response in some of these cell types [21,25].

In the section below, we attempt to compile and reconcile the many studies that have examined the effects of ZIKV NS5 expression and ZIKV infection on various aspects of the host immune response in various cell types and tissue types. We then follow with a discussion on these topics and to explore our hypothesis that the spherical shell-like nuclear structures formed by NS5 in the infected cells can result in different immune modulation within different tissues.

### 3.1. ZIKV NS5 Differentially Modulates the Various Arms of the Host Immune Response

The flavivirus NS5 protein is a well-studied antagonist of the type I IFN response, and ZIKV NS5 is no exception. Multiple studies using both reporter assays and by measuring cellular gene and protein levels, have been performed in different cell types, and they all demonstrate a repression of this pathway (Table 1). While ZIKV NS5 has been shown to degrade STAT2 and block TBK1 activation, all the mechanisms that contribute to this inhibition are still to be elucidated.

We have previously demonstrated that ZIKV NS5 expression alone can induce a cell type-specific transcriptional activation of pro-inflammatory genes in neural LN229 cells, but not in liver Huh7 cells [21]. Many of these pro-inflammatory genes are also upregulated in response to ZIKV infection, but the magnitude of transcriptional activation is much smaller in response to NS5 expression than it is to ZIKV infection [21]. This suggests that ZIKV NS5 can contribute partially to this gene signature. While the detailed mechanism is not known, there is evidence to suggest the involvement of the type II IFN and STAT1-mediated signaling pathways in this pro-inflammatory transcription activation [25]. Further experiments need to be performed to identify the molecular mechanisms involved in this process.

**Figure 3:**
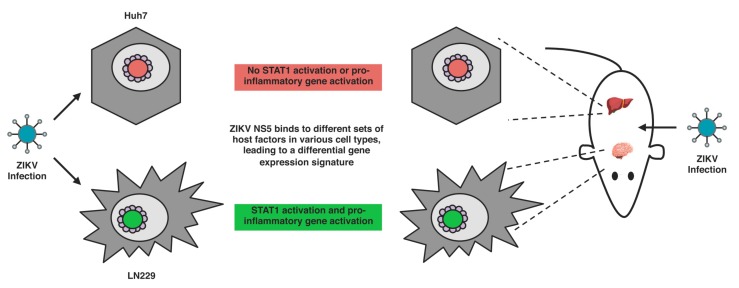
Cell type-specific activation of pro-inflammatory genes by ZIKV NS5. ZIKV infection of cells derived from the brain (LN229) leads to STAT1 activation and pro-inflammatory gene expression. In contrast, ZIKV infection of cells derived from the liver (Huh7) does not trigger any pro-inflammatory response. In an animal model of ZIKV infection, the same pattern of pro-inflammatory gene expression is observed. As NS5′s subcellular localization is the same in both types of cells, it is likely to be interacting with different sets of host factors (green and red circles) in these cells, resulting in the differential gene expression signature observed (Image of organs: Freepik.com).

This stark difference between LN229 and Huh7 transcriptional activation profile is also highly fascinating, as ZIKV NS5 subcellular localization is identical in both cell lines [21] (Figure 3). This suggests that the spherical shell-like nuclear structures of ZIKV NS5 could potentially be interacting with different sets of host proteins during ZIKV infection at different sites of the host. Further experiments to identify the host determinants of the differential transcriptional profiles observed in these different cell types will contribute towards the elucidation of this process.

### 3.2. Role of ZIKV NS5′s Subcellular Localization in Its Modulation of the Host Immune Response

The mechanism by which ZIKV NS5 is able to modulate various arms of the host immune response is not fully elucidated. As discussed in the previous section, ZIKV NS5 has been detected to localize in spherical shell-like structures in the nucleus, despite its known role in virus RNA replication that takes place in RCs in the cytoplasm. But it is not known whether the NS5 proteins that localize to these structures in the nucleus play a role in this cellular function. The ZIKV NS5 NLS mutant protein has a clear and distinct difference in the subcellular localization from the wild-type NS5 protein [21]. When these proteins were separately expressed in neural LN229 cells, the wild-type NS5 protein strongly upregulated expression of many pro-inflammatory genes, while the NS5 NLS mutant induced a much weaker upregulation [21].

This data suggest that ZIKV NS5′s ability to modulate the host immune response can be altered by mutating the NLS sequence and preventing the ZIKV NS5 proteins from entering the nucleus. Hence the ZIKV NS5 proteins that localize in the nucleus appear to be playing a role in the modulation of the host immune response. It is also possible that this observed phenotype is due to the NLS mutation having a direct impact on an interaction with an important host factor or the formation of the spherical shell-like structures. The identification of additional NS5 mutations that alter the subcellular localization of ZIKV NS5 will provide additional tools to study its relationship with its immune modulation function. Interestingly, ZIKV NS5 forms spherical shell-like structures in the nuclei of all known cells, suggesting that the formation of structures alone does not direct the transcriptional response. Hence it is likely that ZIKV NS5 is interacting with separate sets of host factors in the different cell types, and these distinct NS5-host protein interactomes are responsible. The identification of these host proteins will go a long way towards our understanding of these observations. One major caveat of this data at the moment is that these differences are being observed in systems utilizing the overexpression of the ZIKV NS5 protein. Further studies using such a recombinant ZIKV containing these NS5 mutations are necessary to rigorously interrogate this hypothesis in the context of ZIKV infection.

### 3.3. Cell-Type-Specific Pro-Inflammatory Response and Virus Persistence during ZIKV Infection

The host immune response to ZIKV infection has been extensively studied in numerous cell types, and ZIKV infection has been well characterized to antagonize type I IFN signaling (Table 1). On the other hand, we and others have observed that ZIKV infection induces type II IFN signaling and pro-inflammatory gene expression in multiple cell types, including neural and placental cells. In our case, we observed a cell type-specific activation of pro-inflammatory genes when comparing ZIKV infection of liver Huh7 and neural LN229 cells [21] (Figure 3).

As with our studies in Huh7 and LN229 cells, many of the studies showing a pro-inflammatory response to ZIKV infection have been performed in individual cell lines (Table 2). The origin of these cell lines includes the brain, eye, and both male and female reproductive organs. In line with these studies in cell lines, this differential pro-inflammatory response has also been observed in animal models of ZIKV infection [21] (Figure 3), as a pro-inflammatory gene expression signature was only observed in the brain but not the liver of ZIKV infected mice. Early studies in our laboratory also suggest a pro-inflammatory gene signature in the testes. Intriguingly these organs are also sites of persistence and maintenance of viremia in animal models of ZIKV infection as well as studies of humans infected with ZIKV [21,25,98]. This correlation between the persistence of the virus and the activation of pro-inflammatory genes at these sites is notable, and suggests that the transcriptional activation of pro-inflammatory genes is not leading to the clearance of the virus from these sites.

Interestingly, many of these tissues where a pro-inflammatory gene expression signature is observed are also known sites of immune privilege [91,92,99], which allows ZIKV to establish and replicate unimpeded while protected from the peripheral immune system. In addition, the induction of a pro-inflammatory response did not lead to successful clearance of the virus from these sites, and hence it is possible that the pro-inflammatory response might actually be assisting in virus replication and persistence, as has been described for other viruses such as human cytomegalovirus (CMV) and human immunodeficiency virus (HIV) [79,80]. During this process, the virus might be inadvertently causing damage to these sites that become manifested as pathogenesis of virus infection.

Based on the current knowledge in the field, we propose a model in which ZIKV infection induces a pro-inflammatory response in a cell-type-specific matter, and this results in the persistence of the virus and pathological damage at these sites. ZIKV NS5, and its localization into spherical shell-like structures in the nucleus, plays a role in this, possibly by recruiting a cell-type-specific set of host factors to mediate a portion of this pro-inflammatory response (Figure 3). Further studies need to be performed to test this hypothesis, and they will help elucidate how ZIKV infection and the ZIKV NS5 is inducing this organ-specific pro-inflammatory response, and the role of this pro-inflammatory response during ZIKV infection.

In conclusion, the regulation of the subcellular localization of ZIKV NS5, and the role of ZIKV NS5 and infection on the cell-type-specific activation of pro-inflammatory genes are highly complex topics that require detailed analysis in conjunction with reverse genetic and appropriate in vivo model systems. Detailed studies on these topics will help further our understanding of ZIKV infection and perhaps contribute towards the development of antiviral therapeutics and other countermeasures.

## Figures and Tables

**Table 1:** Effects of ZIKV NS5 and infection on host immune responses.

**NS5 Expression**		
**Cell Type**	**Effect**	**Citation**
293/293T	Inhibits ISRE-luc signaling	[67]
	Inhibits ISG54-luc signaling	[26]
	Blocks type I IFN-induced STAT1 phosphorylation	[39]
	Antagonizes type I IFN production	[40]
A549	Antagonizes type I IFN production	[75]
LN229	Activates pro-inflammatory genes	[21]
**ZIKV Infection**		
**Cell Type**	**Effect**	**Citation**
DCs	Inhibits type I IFN signaling	[76]
293T	Inhibits type I IFN signaling	[26]
Dendritic cells	Inhibits type I IFN induced STAT1 and STAT2 phosphorylation	[75]
JEG3 SF268	Activates STAT1-mediated type II IFN and pro-inflammatory pathways	[25]
LN229	Induces STAT1 phosphorylation and activation of pro-inflammatory genes	[21]
Brain Microglia	Induces inflammation	[77]
Retina Pigment Epithelial	Induces inflammatory response	[76]

**Table 2:** Summary of organs in which ZIKV infection leads to an inflammatory response and/or pathology in cell culture (grey), animal models (green) and human samples (blue). Y denotes a positive finding while N means it was not demonstrated in the cited paper.

Organ	Cell/Tissue Type	Inflammatory Response	Pathology	Citation
Brain	SF268	Y	N	[25]
	LN229	Y	N	[21]
	Brain Microglia	Y	N	[77]
	NPCs	N	Y	[93]
	NPCs	Y	N	[81]
	Neurospheres/Organoids	N	Y	[94]
	Brain	N	N	[86]
	Brain	N	N	[95]
Eye	Retina Pigment Epithelial	Y	N	[76]
	Eye	N	N	[82]
	Conjunctival fluid	N	N	[88]
Male Reproductive Tract	Sertoli	N	N	[96]
	Testis	Y	Y	[85]
	Testis	N	N	[86]
	Testis	N	Y	[84]
	Sperm/Semen	N	N	[91,92]
Female Reproductive Tract	JEG3	Y	N	[25]
	Uterine fibroblasts	N	N	[28]
	Ovary	Y	N	[97]
	Cervical mucus	N	N	[89]
	Vagina secretions	N	N	[90]
	Placenta	N	N	[95]
